# Single cigarette sales contravene tobacco control policies, Brazil

**DOI:** 10.2471/BLT.24.291807

**Published:** 2024-10-02

**Authors:** André Salem Szklo, Cristina de Abreu Perez, Letícia Casado, Érica Cavalcanti, Felipe Lacerda Mendes

**Affiliations:** a Tobacco Control Unit, Instituto Nacional de Câncer, Rua Marquês de Pombal 125/7 andar 20230-240 Centro, Rio de Janeiro, Brazil.; b Executive Secretariat of the National Commission for WHO FCTC Implementation, Instituto Nacional de Câncer, Rio de Janeiro, Brazil.; c Education and Teaching Coordination, Instituto Nacional de Câncer, Rio de Janeiro, Brazil.

On 27 October 2005, its Senate ratified Brazil’s adherence to the World Health Organization (WHO) Framework Convention on Tobacco Control (WHO FCTC).^1^ Since then, WHO FCTC measures have become the National Policy on Tobacco Control. In 2019, WHO recognized Brazil as the second country in the world to implement, at the highest level, the main measures of the MPOWER policy package, which consists of six evidence-based measures to reduce the demand for – and supply of – tobacco.^1^ However, non-compliance with existing anti-tobacco laws aimed at preventing smoking initiation, such as the ban on the sale of single cigarettes, the regulation of retail points of sale and the strict prohibition of the sale of tobacco products to minors (that is, younger than 18 years), remains a significant obstacle to reducing the proportion of adolescent smokers, estimated at almost 7% in 2019.^2^^–^^4^

In Brazil, the government considers the illegal sale of single cigarettes both an economic and sanitary infraction.^5^^,^^6^ According to the regulations of the Secretariat of Federal Revenue regarding the tax levied on manufactured products, cigarettes may only be sold in closed packages containing 20 units. Additionally, the National Health Regulatory Agency grants registration for a manufacturer’s brand only after reviewing a sample of the product’s packaging intended for commercialization, and also regulates the printing of health warnings on these packages.

The affordability of single cigarettes is an economically advantageous option to start and/or continue smoking, particularly for young people and/or individuals with limited financial resources.^4^^,^^7^ Additionally, the cigarette stick is used by tobacco companies for communicative purposes, intending to facilitate brand differentiation and influence smokers’ perceptions of brand attributes.^8^ Not surprisingly, the tobacco industry exerted pressure against a nationwide ban on single cigarettes in Uganda,^9^ one of the many African countries where the relative size of the adult consumer base for single cigarettes is high.^7^ Even in countries with different patterns of single cigarette consumption among the overall population, such as Brazil,^2^^,^^10^ their affordability and the lack of the legal provisions for warning labels contribute to smoking initiation among youth. As adolescents grow up and become more addicted, the tobacco industry benefits from their continued purchase of cigarette packs rather than single cigarettes.^2^^,^^10^

In Brazil, the ongoing availability of single cigarettes as a means of purchase raises concerns.^2^ The strengthening of tax policy between 2008 and 2013 was accompanied by an increase in the proportion of single cigarette purchases.^2^ Despite the decline in the real price of cigarette packs since 2017, approximately one in five smokers of manufactured cigarettes aged between 18 and 24 years in Brazil reported buying single cigarettes in their last purchase in 2019, compared to less than one in 10 among older people.^2^ Worryingly, a study in 2019 on the low compliance with the ban on the sale of cigarettes to minors found that, among adolescents who successfully and regularly purchased cigarettes illegally in legally authorized commercial establishments, 70% (1612/2288) reported that buying single cigarettes was the most common method.^3^ The purchase occurred in bars, newsstands and grocery stores, thereby also increasing the value tobacco companies may give to sticks for communicative purposes in Brazil. A comparison with data from the Global Youth Tobacco Survey, collected between 2010 and 2018 in 140 countries and territories, reveals that this figure is significantly higher in Brazil compared to other countries in the WHO Region of the Americas (41%) and the global average (37%).^11^

Researchers involved in the study Tobacco Industry Advertising and Sale Strategies Around Public Schools in Five Brazilian Capitals^12^ inspected various points of sale across the country from March to August 2023 to assess compliance with existing anti-tobacco laws. From each of the five Brazilian regions, the state capital with the highest smoking prevalence among individuals aged 13–17 years was selected. In each selected city, one public school was chosen based on criteria established by the researchers rather than by statistical sampling methods, as follows: the school had to be open during daytime; located in a highly populated, commercial non-residential area frequented by students from varying socioeconomic backgrounds; and had to have students aged 13–17 years who were allowed to walk alone near the school. Using these schools as reference points, a radius of 300 m was established to map the sales reach.

Among the 170 legally authorized commercial establishments identified, 82 (48%) sold tobacco products. Of these 82 licensed outlets, 71 (87%) were observed selling single cigarettes. Our findings highlighted the affordability of single flavoured cigarettes to adolescents, typically priced between 0.10 and 0.40 United States dollars (US$), whereas a pack containing 20 cigarettes of the most-sold brand costs around US$ 1.00. In licensed establishments, these products were often strategically placed near sweets and candies, which is also illegal according to Brazil’s regulation.^6^ Sometimes, single cigarettes were the sole available means of purchase, with a note on the display stating, “I do not sell cigarette packs, only single cigarettes. Please do not insist!”

Whether tobacco companies are involved in or have any knowledge of these illicit practices in Brazil is unclear. However, the use of similar advertising and promotional strategies at points of sale to target children and youth worldwide suggests that multinational tobacco companies might be engaged in infringing existing laws and regulations.^4^^,^^9^ Tobacco retailers most likely do not have the motivation or capability to share their cigarette promotion practices with counterparts in other countries, as opposed to multinational tobacco companies, which have a vested interest in and considerable resources to control how retailers market their products globally. Adolescents are indeed the primary targets of tobacco industry marketing strategies, which aim to replace adult smokers who either quit or die;^10^ in Brazil, the tobacco industry also benefits from the fact that, as people age and/or develop nicotine dependence, they tend to smoke more cigarettes per day, leading to increased purchases of cigarette packs over single cigarettes.^2^^,^^10^

The availability of single cigarettes should encourage authorities to undertake educational and enforcement measures. For instance, tobacco control governmental agencies should raise awareness and provide training to local health surveillance and consumer protection agents, enabling them to incorporate the monitoring of single cigarette sales into their inspection routines at points of sale.^1^ This effort also requires collaboration with retail trade organizations and unions representing various commercial sectors. Although the sale of single cigarettes creates a perverse incentive for retailers to seek additional profits, Brazil has already demonstrated a successful example of combining societal mobilization with legislative enforcement to reduce smoking prevalence, such as the effective implementation of the smoke-free law, a regulation that protects individuals from exposure to environmental tobacco smoke in enclosed public spaces.^1^^,^^6^ However, recognizing and combating the increasing and successful interference by the tobacco industry in Brazil against anti-tobacco policies is also crucial. For example, the interference that blocked the implementation of the ban on the sale of flavoured tobacco products in the Brazilian market.^13^

Since retailers who sell single cigarettes may also be more likely to circumvent other tax laws,^2^^,^^3^ such as the sale of brands not approved on the Brazilian market by the Health Regulatory Agency,^5^^,^^6^^,^^13^ our findings could create opportunities for the health sector in Brazil to more assertively strengthen WHO FCTC multisectoral measures through the full implementation of article 15 of the WHO FCTC and the Protocol to Eliminate Illicit Trade in Tobacco Products.^1^ Additionally, to make the law more effective, understanding through qualitative and quantitative studies the various mechanisms young people use to circumvent the law prohibiting the sale of single cigarettes is important.^2^ Such mechanisms include purchasing from smaller establishments and always from the same place. 

Moreover, the National Commission for WHO FCTC Implementation should prompt the Public Prosecutor’s Office to initiate negotiations with tobacco companies that supply the extensive network of retailers nationwide to establish a conduct adjustment term. Multinational tobacco companies have both a strong interest in and ample resources to influence or even control how retailers advertise and promote their products.^1^^,^^4^ Thus, in such agreements, companies should commit to a shared responsibility for enforcing laws prohibiting the sale of cigarettes to minors and the sale of single cigarettes. At the same time, mobilizing members of the legislative branch to propose and pass a federal law restricting the sale of tobacco products to licensed tobacco stores is important – as opposed to selling them in bars, newsstands and grocery stores where tobacco products can currently be sold alongside other goods.

Finally, our findings also offer the country a learning opportunity for implementing new policies. For instance, they highlight the need to adopt strategies to make the cigarette stick less attractive, such as manufacturing it in a plain and standardized form and/or displaying health messages and explicit warnings.^8^

The continuous sale of single cigarettes,^2^ along with its negative implications for tobacco taxation and communication policies,^7^^,^^8^ contributes to the increasing health, economic and social burden of tobacco, particularly in low- and middle-income countries.^13^ Brazil has arguably one of the most scrutinized tobacco control policy regimes, and this article offers countries a learning opportunity to improve their tobacco control policies.
